# What Should I Eat before Exercise? Pre-Exercise Nutrition and the Response to Endurance Exercise: Current Prospective and Future Directions

**DOI:** 10.3390/nu12113473

**Published:** 2020-11-12

**Authors:** Jeffrey A. Rothschild, Andrew E. Kilding, Daniel J. Plews

**Affiliations:** Sports Performance Research Institute New Zealand (SPRINZ), Auckland University of Technology, Auckland 0632, New Zealand; andrew.kilding@aut.ac.nz (A.E.K.); daniel.plews@aut.ac.nz (D.J.P.)

**Keywords:** cycling, running, carbohydrate, adaptations, periodization, fasting

## Abstract

The primary variables influencing the adaptive response to a bout of endurance training are exercise duration and exercise intensity. However, altering the availability of nutrients before and during exercise can also impact the training response by modulating the exercise stimulus and/or the physiological and molecular responses to the exercise-induced perturbations. The purpose of this review is to highlight the current knowledge of the influence of pre-exercise nutrition ingestion on the metabolic, physiological, and performance responses to endurance training and suggest directions for future research. Acutely, carbohydrate ingestion reduces fat oxidation, but there is little evidence showing enhanced fat burning capacity following long-term fasted-state training. Performance is improved following pre-exercise carbohydrate ingestion for longer but not shorter duration exercise, while training-induced performance improvements following nutrition strategies that modulate carbohydrate availability vary based on the type of nutrition protocol used. Contrasting findings related to the influence of acute carbohydrate ingestion on mitochondrial signaling may be related to the amount of carbohydrate consumed and the intensity of exercise. This review can help to guide athletes, coaches, and nutritionists in personalizing pre-exercise nutrition strategies, and for designing research studies to further elucidate the role of nutrition in endurance training adaptations.

## 1. Introduction

From Olympians to recreational exercisers, athletes of all levels face the same questions—what should I eat before exercise, and how does it affect my training? Despite being relevant to anyone performing exercise, many questions relating to the effects of nutritional intake on endurance training responses and adaptations remain unanswered.

The duration and intensity of exercise are the most important factors influencing the adaptive response to endurance training [1]. However, strategies altering nutrient availability before and during exercise can also impact training adaptations by modulating the exercise stimulus and/or cellular responses to the exercise-induced perturbations [2]. Specific strategies to alter nutrient availability can include exercising in the overnight-fasted state, restricting carbohydrate (CHO) ingestion between training sessions, and increasing CHO ingestion before or during exercise [3]. Although performance may be improved following pre-exercise CHO ingestion [4,5], exercise undertaken with reduced availability of CHO can increase the activation of key signaling proteins compared with exercise performed with high CHO availability [6], potentially influencing longer-term training adaptations.

**Figure 1:**
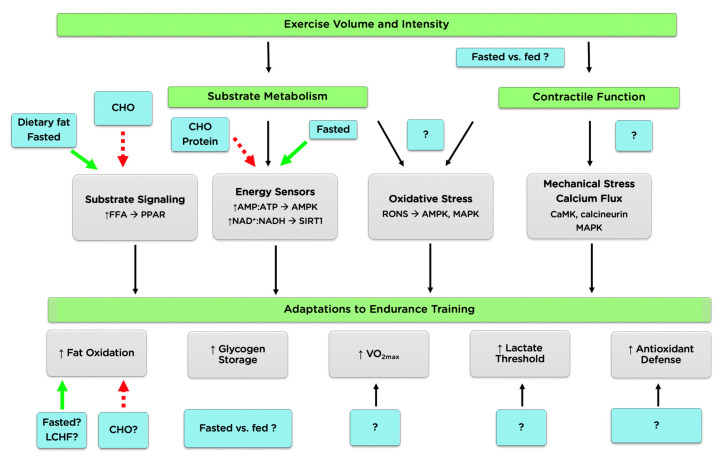
Schematic of areas where pre-exercise nutrition has the potential to impact the adaptive responses to endurance training. Green arrows suggest the potential to increase or augment specific signaling, and red dashed arrows suggest the potential to decrease or impair specific signaling. Abbreviations: AMPK, AMP-activated protein kinase; CaMK, calcium/calmodulin-stimulated protein kinase; CHO, carbohydrate; FFA, free fatty acids; LCHF, low-CHO high-fat; MAPK, mitogen-activated protein kinase; VO_2max_, maximal oxygen consumption.

Among the intracellular signals comprising the endurance training response are mechanical stretch, reactive oxygen and nitrogen species (RONS), calcium flux, AMP:ATP ratio, and the availability of endogenous CHO and free fatty acids (FFA) [7,8]. These signals are affected by both the duration and intensity of an exercise session, and by the pre-exercise nutrition choices of an athlete (i.e., the size, type, and timing of the pre-exercise meal(s), Figure 1). Although some lines of evidence suggest ingesting CHO before exercise can negatively influence endurance training adaptations, contrasting findings have been reported. For example, ingesting CHO has decreased [9], increased [10], or had no effect [11] on the activity of the 5′ AMP-activated protein kinase (AMPK) following exercise. Similarly, training-induced improvements in maximal oxygen consumption (VO_2max_) have been reported to increase [12], decrease [13], or remain unchanged [14] following 4–6 weeks of CHO-fed compared with fasted-state training. These contrasting findings can be a source of confusion and may explain why the beliefs and practices relating to the role and influence of pre-exercise nutrition vary so widely among coaches and athletes [15,16]. Accordingly, the purpose of this review is to highlight the current knowledge of the influence of pre-exercise nutrition ingestion on the metabolic, physiological, and performance responses to endurance training. We also highlight areas for practitioners where evidence is lacking, particularly regarding trained athletes, and suggest directions for future research.

## 2. Acute Responses to Pre-Exercise Nutrition Intake

The vast majority of pre-exercise nutrition interventions have been conducted in an acute context. Although acute responses to training do not always correspond with long-term adaptations [17,18], the accumulation over time of transient, exercise-induced changes in gene expression are thought to be the driving factor behind many adaptations to training [19]. Therefore, it is relevant to consider the acute effects of pre-exercise nutrition in addition to the longer-term adaptations.

### 2.1. Metabolism and Substrate Oxidation

The liver plays a key role in metabolic regulation during extended exercise [20]. Despite the ~40% reduction in liver glycogen following an overnight fast [21], blood glucose concentration can be maintained at normal levels during exercise due to increased gluconeogenesis and/or decreased utilization of glucose in skeletal muscle [22,23]. However, fatigue during extended exercise is often associated with reduced blood glucose concentrations [24], supporting a critical role for liver glycogen in achieving optimal performance during extended exercise.

Exercising in the fasted-state generally allows higher levels of fat oxidation than exercise performed in the CHO-fed state during low-to-moderate intensity exercise [25] and can increase the relative intensity where maximal fat oxidation occurs [26]. Ingesting CHO before exercise increases plasma glucose and insulin levels, leading to a reduction in hepatic glucose output and an increase in skeletal muscle glucose uptake during exercise [27]. This can lower fat oxidation by decreasing plasma FFA availability via insulin-mediated inhibition of lipolysis [28], and also by inhibiting fat oxidation within the muscle due to an increased glycolytic flux [29]. Intramuscular triglycerides (IMTG) provide a key substrate for fat oxidation, primarily during exercise in the fasted state [30,31], although their use declines as the duration of exercise extends, while the oxidation of plasma FFA increases [32]. Up to 6 h may be required following a CHO-rich meal for substrate oxidation and glucose homeostasis to return to levels observed during fasted-state exercise [33].

In contrast with exercise performed in the overnight-fasted state, which lowers hepatic but not muscle glycogen [34], restricting CHO between training sessions allows exercise to be undertaken with reduced muscle glycogen concentrations [35]. During exercise with low muscle glycogen there is an increase in the oxidation of fat [36,37] and amino acids [38,39], and a reduction in muscle glycogen breakdown [36,40,41]. During exercise undertaken with normal muscle glycogen levels, muscle glycogen breakdown is similar between fed and fasted-state exercise [31,42,43,44] and may be reduced when ingesting CHO during exercise [45].

The majority of research looking at fat oxidation has compared CHO to a placebo, but the use of pre-exercise protein ingestion represents an interesting and under-researched area. Consumption of protein before and during steady-state exercise did not affect FFA availability or whole body fat oxidation compared with fasted-state exercise commenced with normal [46] or lowered [47] muscle glycogen concentration, despite elevated insulin levels. This may be related to the increases in catecholamine levels during exercise, which are an important determinant of the adipose tissue lipolytic rate and can override the inhibition by insulin [48]. Although protein ingestion before exercising in a low-glycogen state has no effect on rates of muscle protein synthesis, it is plausible that it could reduce muscle protein breakdown during exercise [49]. It also appears possible that pre-exercise protein ingestion increases amino acid oxidation during exercise [49], but further quantification of its influence is needed.

**Figure 2:**
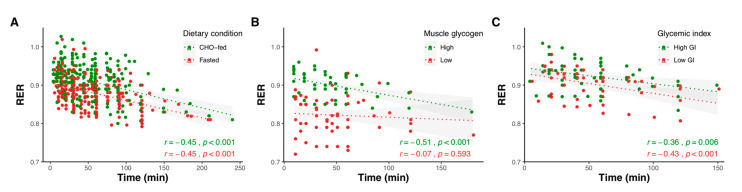
Substrate oxidation in relation to exercise duration for studies reporting respiratory exchange ratio (RER) at multiple time points comparing overnight-fasted and/or CHO-fed exercise with normal muscle glycogen levels (**A**), exercise undertaken with high (471 ± 208 mmol kg^−1^ dry mass) and low (232 ± 112 mmol kg^−1^ dry mass) muscle glycogen levels (**B**), and following high (82 ± 10) and low (36 ± 9) glycemic index meals (**C**). Shaded areas represent 95% confidence intervals. Data were obtained by pooling results from 60 studies (see supplementary files for references).

**Figure 3:**
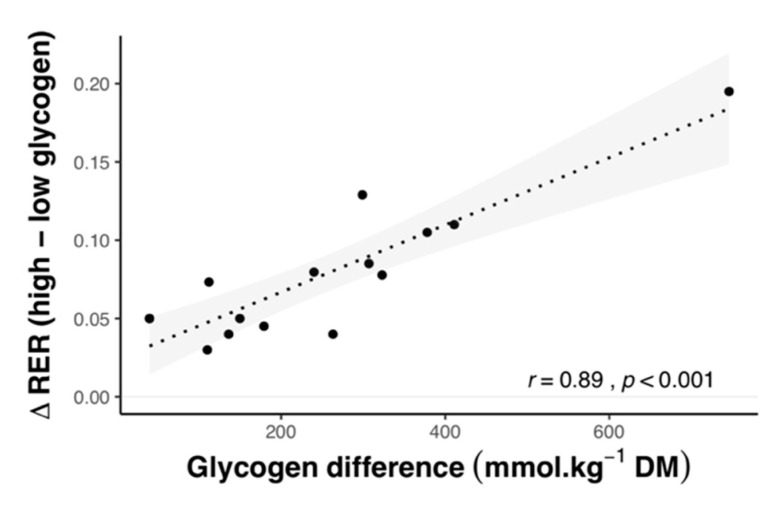
Correlation between differences in respiratory exchange ratio (RER) during exercise and differences in pre-exercise glycogen levels. Shaded area represents 95% confidence intervals. Data were obtained by pooling results from 13 studies that manipulated glycogen levels and reported RER for high- and low-glycogen trials (see supplementary files for references). DM = dry mass.

**Figure 4:**
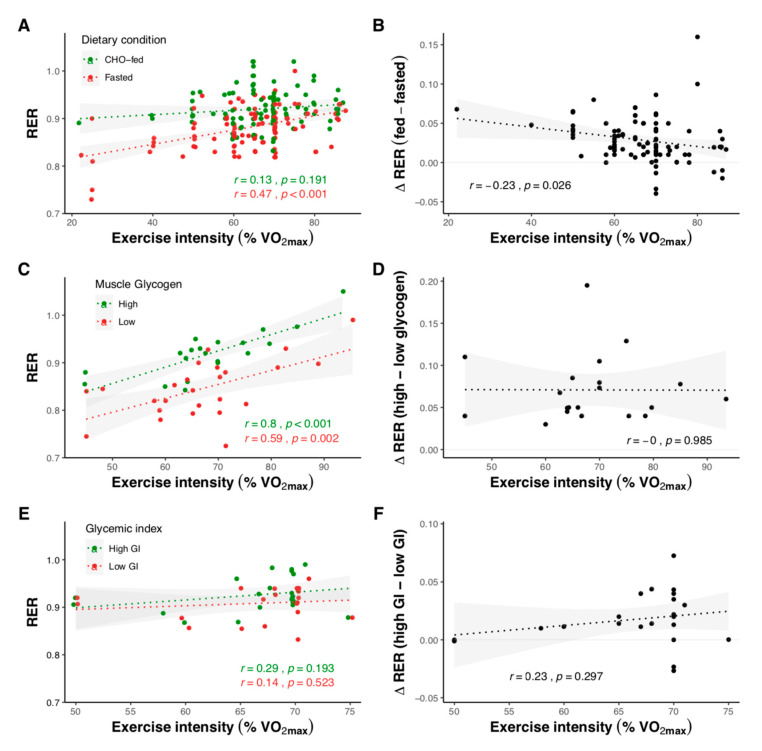
Substrate oxidation in relation to exercise intensity for studies comparing overnight-fasted and CHO-fed exercise with normal muscle glycogen levels (**A**,**B**), exercise undertaken with high (471 ± 208 mmol kg^−1^ dry mass) and low (232 ± 112 mmol kg^−1^ dry mass) muscle glycogen levels (**C**,**D**), and following high (82 ± 10) and low (36 ± 9) glycemic index meals (**E**,**F**). Shaded areas represent 95% confidence intervals. Data were obtained by pooling results from 103 studies (see supplementary files for references).

**Figure 5:**
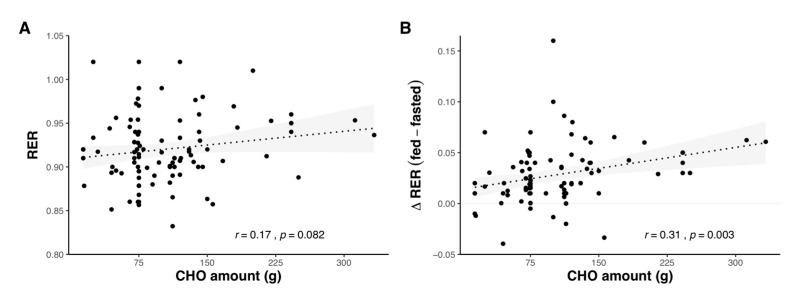
Substrate oxidation in relation to amount of carbohydrate (CHO) consumed before exercise, as absolute RER value during exercise (**A**) and difference in RER between fed and fasted-state exercise (**B**). Shaded areas represent 95% confidence intervals. Data were obtained by pooling results from 76 studies (see supplementary files for references).

**Figure 6:**
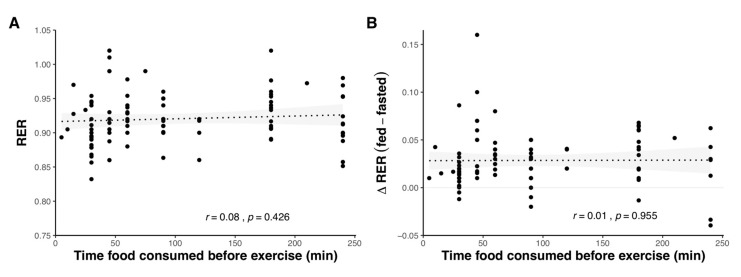
Substrate oxidation in relation to the time food was consumed before exercise, as absolute RER value during exercise (**A**) and difference in RER between CHO-fed and fasted-state exercise (**B**). Shaded areas represent 95% confidence intervals. Data were obtained by pooling results from 76 studies (see supplementary files for references).

**Figure 7:**
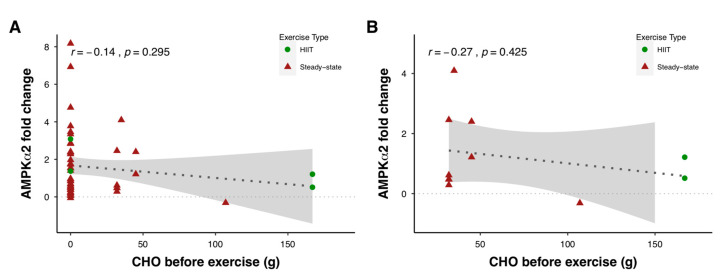
Relationship between AMPKα2 activity during exercise (measured as fold-change from pre-exercise resting levels to immediately post-exercise) and carbohydrate (CHO) intake before exercise including (**A**) and excluding (**B**) studies that tested in the overnight-fasted state. HIIT: high-intensity interval training. Shaded areas represent 95% confidence intervals. Data were obtained by pooling results from 22 studies (see supplementary files for references), which included 265 participants (6.0% female), 25.1 ± 2.8 years, VO_2max_ 52.9 ± 11.0 mL kg^−1^ min^−1^.

To compare the influence of pre-exercise CHO ingestion, muscle glycogen levels, and glycemic index on substrate oxidation and AMPK activity, we pooled the results of 125 studies (available as supplementary online files) that included the relevant intervention groups (Figure 2, Figure 3, Figure 4, Figure 5, Figure 6 and Figure 7). Together, these studies included 1245 subjects (12.8% female), with an average age, BMI, and VO_2max_ of 25.4 ± 3.1 years, 23.2 ± 1.4 kg m^2^, and 56.7 ± 8.2 mL kg^−1^ min^−1^. Linear correlation analysis was used to calculate the correlation coefficient between variables, according to Pearson’s product moment (r) using R statistical software. Pooled data are reported as mean ± SD, with the level of statistical significance set at *p* < 0.05.

#### 2.1.1. Effect of Exercise Duration

The respiratory exchange ratio (RER—a measure of substrate oxidation) decreases with exercise duration, indicating an increasing reliance on fat oxidation as the duration of exercise extends [50]. Differences in RER between exercising in the fed vs. fasted state and following low vs. high glycemic index CHO remain largely similar throughout exercise, while the differences in RER between high and low starting muscle glycogen decrease as exercise duration extends (Figure 2). The latter could presumably be related to the greater utilization of muscle glycogen during exercise undertaken with higher levels of glycogen, leading to more similar levels during the later stages of exercise. This idea is supported by the pooled data, which show a strong correlation (r = 0.89, *p* < 0.001) between the differences in pre-exercise glycogen levels and differences in RER during exercise (Figure 3).

#### 2.1.2. Effect of Exercise Intensity

Exercise intensity is well-established to influence substrate oxidation during exercise, with RER increasing with intensity [51]. Differences in RER between fed and fasted-state exercise are larger at lower intensities and decrease as intensity increases (Figure 4A,B). In contrast, exercise undertaken with low muscle glycogen maintains lower RER values compared with normal glycogen, despite increasing exercise intensity (Figure 4C,D). The glycemic index of the pre-exercise meal appears to have minimal effects on the relationship between intensity and substrate oxidation (Figure 4E,F).

#### 2.1.3. Effect of Carbohydrate Amount

Several studies have directly compared varying amounts of CHO ingested before exercise, either showing no differences in substrate oxidation with varying amounts of pre-exercise CHO [5,52,53,54], or differences throughout all [55] or portions [56,57] of the exercise bout. When pooling a number of studies together, there is a weak positive relationship between the amount of CHO ingested and RER during subsequent exercise, while differences in RER between CHO-fed and fasted-state exercise increase as the amount of CHO ingested is increased (Figure 5).

#### 2.1.4. Effect of Pre-Exercise Meal Timing

The amount of time before exercise food is consumed is another factor that can influence metabolism and substrate oxidation, and studies have undertaken exercise in the fed state between 5 [58] and 240 min [59,60] post-prandial. Although direct comparisons of the influence of meal-timing are limited, no differences in substrate oxidation were found when the same meals were ingested 15, 45, or 75 min [61] and 30, 60, or 90 min [62] before exercise. When consumed within 4 h of exercise, the amount of time prior to exercise does not have a meaningful impact on substrate oxidation (Figure 6).

#### 2.1.5. Summary and Future Directions

During submaximal steady-state exercise, fat oxidation is generally higher in the overnight-fasted compared with CHO-fed state. Although fat oxidation increases with exercise duration, fasted-state exercise increases fat burning throughout the duration of exercise compared with consuming CHO before exercise. However, as exercise intensity increases the difference in fat oxidation between CHO-fed and fasted-state exercise diminishes. Fat oxidation is also higher when undertaking exercise with low, compared with normal muscle glycogen levels, with the differences maintained across varying exercise intensities but diminishing as the duration of exercise extends. While the amount of time before exercise food is consumed does not meaningfully influence substrate oxidation, greater amounts of CHO in the pre-exercise meal leads to greater differences in substrate oxidation between fed and fasted-state exercise. These findings are most applicable to moderately-trained males, who made up ~87% of study participants. Substrate metabolism may differ between males and females [63], with differences further affected by the female menstrual cycle [64] and the use of oral contraceptives [65]. Additionally, sedentary populations typically show no differences in post-exercise glucose, insulin, or FFAs between fasted and fed conditions [66], which is in contrast with trained athletes [67,68,69] who also show a greater capacity for fat oxidation compared with untrained or recreationally active populations [70].

Despite fasted-state training being performed by a large number of endurance athletes [15], there are potential negative implications from its use. Particularly for athletes doing a high volume of training, exercising in the overnight-fasted state could more likely lead to a negative energy balance, which can be associated with hormonal and immune dysfunction [71]. As a method of providing energy intake while still allowing higher levels of fat oxidation, future studies should examine the effects of a protein-rich breakfast on fat oxidation during exercise, in direct comparison with exercise following a CHO-rich breakfast and in the overnight-fasted state. As this approach is currently utilized by few endurance athletes [16], it could be a useful tool for those who want to increase fat burning without incurring a large caloric deficit. The influence of various pre-exercise meals on gut comfort should also be investigated, as a large number of athletes perform fasted-state training to avoid gut discomfort [15]. Exercise-induced gastrointestinal distress is beyond the scope of this review but has been reviewed elsewhere [72,73].

### 2.2. Cell Signaling

Among the key intracellular signals influencing skeletal muscle adaptations to endurance training are changes in the AMP:ATP ratio, contraction-induced changes in mechanical strain, increased calcium flux, an increase in RONS, and the availability of endogenous CHO and FFA [7,8,74]. Nutritional intake has the potential to modify signaling across several of these pathways, primarily related to energy sensing and nutrient availability.

#### 2.2.1. Energy Sensing and the AMP-Activated Protein Kinase

The 5′ AMP-activated protein kinase (AMPK) is a cellular energy sensor that regulates cellular and whole-body energy balance by inhibiting ATP-consuming pathways and activating ATP-producing pathways [75]. Activation of AMPK can lead to a range of metabolic adaptations including increases in glucose uptake, glycolytic flux, fat oxidation, and mitochondrial biogenesis [76]. The degree of AMPK activation during exercise can be influenced by exercise intensity [77], training status [78], muscle glycogen [79], and nutrient availability [80].

When starting exercise with normal muscle glycogen levels, studies that have shown a blunting effect of CHO ingestion on AMPK-α2 activity [9,81] have been at lower intensities than those showing no differences between CHO-fed and fasted-state exercise [11,82]. Conversely, exercise that is undertaken with low, compared with normal muscle glycogen levels, has resulted in greater increases in the activity of AMPK-α2 following 1 h of steady-state endurance exercise at 65–70% VO_2max_ [83,84,85], but similar increases in AMPK activity and/or phosphorylation were seen following both exhaustive and non-exhaustive high-intensity exercise undertaken with high and low muscle glycogen levels [86,87,88]. Therefore, ingesting CHO before exercise may dampen AMPK activity during low but not high-intensity exercise, and an intensity threshold may exist below which CHO ingestion could blunt AMPK signaling.

The CHO content of the pre-exercise meal size could also influence molecular signaling. Compared with exercising in the fasted state, consumption of <70 g CHO prior to exercise had no effect [11,82] or even increased [10] skeletal muscle AMPK signaling following exercise compared with exercise performed in the fasted state. In contrast, ingesting 130–160 g of CHO before exercise reduced the exercise-induced increases in AMPK^Thr172^ phosphorylation [89], with the phosphorylation of acetyl-CoA carboxylase (ACC) decreased [36] or unaffected [89]. When pooling a number of studies together, non-significant correlations can be observed between the exercise-induced increases in AMPK-α2 activation and CHO intake before exercise (Figure 7). Future studies that are designed to examine the relationships between meal size, exercise type and intensity, and AMPK activity are warranted.

Interpretation of the research comparing pre-exercise nutrition choices on AMPK activity during exercise is complicated by the small number of studies available, training status of participants, and specific markers being reported. For example, AMPK-α2 activity during exercise is reduced by short- and longer-term endurance training, making it difficult to compare between trained and untrained subjects [78,90,91]. Additionally, some studies report the phosphorylation of AMPK^Thr172^, which reflects phosphorylation of both AMPK-α1 and -α2 subunits and may be less sensitive for detecting changes in AMPK activity that are only occurring in the -α2 subunit that is more responsive to exercise [81,82,86]. Further complicating interpretation of the available literature, several studies have shown a blunting effect of CHO ingestion on AMPK-α2 activity or AMPK^Thr172^ phosphorylation, yet similar increases in phosphorylation of ACC, a downstream substrate of AMPK [81,83,92]. Similar increases in PGC-1α mRNA expression following HIIT performed with low or high CHO availability have also been reported, despite phosphorylation of ACC being reduced by high CHO availability [36,93]. Furthermore, despite an attenuation of exercise-induced AMPK activation when ingesting CHO during a single bout of exercise [81], no differences in training adaptations were observed following 10 weeks of training with or without CHO ingestion during exercise [94]. These apparent discrepancies could be due to crosstalk between signaling pathways and/or the wide variability in exercise-induced changes in mRNA expression [95] and highlight the importance of looking at longer-term changes in mitochondrial content or function rather than acute changes in specific proteins.

#### 2.2.2. Contraction-Induced Signaling

Another key intramuscular signal comes from increased calcium released during muscle contraction. Calcium-dependent transcriptional pathways play important roles in regulating fat oxidation, mitochondrial biogenesis, and muscle fiber-type changes via myocyte enhancer factor 2 (MEF2) and p38 mitogen-activated protein kinase (MAPK) [96,97,98,99]. Few studies have compared the effects of nutrition interventions on calcium-dependent, contraction-induced signaling pathways. There appear to be minimal effects of exercise performed in the fed vs. fasted-state or with varying levels of muscle glycogen [36,87,89,100], but some evidence suggests p38 may be sensitive to nutrient status [101,102]. Although more research is needed, the independence of these pathways from nutritional influence could help to explain why similar longer-term changes could be observed when training under differing nutritional conditions.

#### 2.2.3. Substrate Signaling

Exercise performed in the overnight-fasted state generally results in higher levels of FFA compared with CHO-fed exercise, and an inverse relationship is seen between FFA concentration and CHO oxidation during exercise [33]. In addition to acting as substrate for β-oxidation in the mitochondria, FFA also play a role in molecular signaling cascades that regulate fatty acid metabolism and mitochondrial biogenesis, via activation of peroxisome proliferator-activated receptors (PPAR), MAPKs, and sirtuin 1 [7,103,104,105]. Some studies have found differences in FFA between fed and fasted state throughout an entire bout of exercise [50,106], while others have shown differences appearing from 20 [59], 30 [107], 45 [4], or 60 min [108] into exercise. These differences do not appear to show any pattern related to meal size, time of ingestion, or exercise intensity. Similar levels of FFA are found during exercise in the fasted-state and following ingestion of a high-fat meal [60,109] or following pre-exercise protein ingestion with normal [46] and low [47] muscle glycogen levels. Although a high-fat diet, in the absence of exercise, can increase rates of fat oxidation during exercise, a high-fat intake by itself does not increase mitochondrial content or exercise performance without simultaneously engaging in exercise training [105]. Future studies are needed to determine if differences in FFA during CHO-fed vs. fasted-state can significantly alter training adaptations.

#### 2.2.4. Reactive Oxygen and Nitrogen Species

Rather than simply being a byproduct of oxidative stress, RONS play a direct role in regulating the response to both acute exercise (e.g., muscle contractile function, glucose uptake, blood flow, and cell bioenergetics) and longer-term exercise training (e.g., mitochondrial biogenesis, muscle hypertrophy, angiogenesis, and redox homeostasis) [110]. Very little research exists looking at the influence of a pre-exercise meal on the oxidative stress response to a bout of exercise. At rest, a high-CHO meal can evoke a greater postprandial oxidative stress response compared with a high-fat meal [111], while the addition of olive oil to a meal reduced post-meal increases in oxidative stress markers, such as NADPH oxidase and 8-isoprostane, both of which have been associated with endurance training adaptations [112,113,114]. Acute and chronic fruit ingestion can dampen lipid oxidation during exercise [115], and fruit-derived phenolic compounds may promote muscle fiber-type transformation [116]. Whey protein can also impact the antioxidant defense system by enhancing activity of the endogenous antioxidant enzymes [117]. It is currently unknown how various pre-exercise meals affect oxidative stress in response to exercise and if there are any longer-term training implications.

#### 2.2.5. Summary and Future Directions

Overall, it appears that ingesting small amounts of CHO (<75 g) does not meaningfully impair mitochondrial signaling, but lower-intensity exercise may be more influenced by CHO ingestion than high-intensity exercise. Beyond the differences in exercise intensity and duration, interpretation of the existing literature is further challenged by several studies comparing the effects of fasted and CHO-fed exercise that have provided CHO both before and during exercise [9,30,118,119]. This is relevant because CHO ingestion during exercise can reduce muscle glycogen breakdown [45], which itself may be a key signal for AMPK activity [79] and alter levels of TCA cycle intermediates [120].

Although crosstalk between signaling pathways exists, higher-volume endurance training is more likely to influence training adaptations through the contraction-induced signaling pathways, while higher-intensity training, which increases the AMP:ATP ratio, appears more likely to signal for mitochondrial biogenesis through energy-sensing pathways [121]. It is possible that there may be a threshold for the amount of CHO ingested before exercise (~75 g), above which may impair intracellular (e.g., AMPK) signaling, independent of muscle glycogen levels. This is relevant as a large number of endurance athletes report consuming a small amount of CHO-based foods before training [15]. It is also possible that the influence of CHO ingestion on AMPK signaling may be related to exercise intensity. Future research could seek to better understand the interplay between exercise intensity and the amount of CHO ingested before and/or during exercise, bearing in mind that interactions between CHO ingestion and exercise intensity may be different during continuous and intermittent exercise [122]. Additionally, a better understanding of the influence of pre-exercise nutrition on RONS signaling during exercise is needed.

### 2.3. Performance

Pre-exercise CHO ingestion has been found to generally enhance prolonged (>60 min), but not shorter duration aerobic exercise performance [66]. However, ingesting CHO during exercise minimizes the differences between consuming CHO or a placebo prior to exercise [123,124,125,126]. The vast majority of studies comparing performance in the fed or fasted state have used steady-state endurance exercise [66], but similar effects of exercise duration are found with HIIT, as performance was improved in the fed state for 90 min of high-intensity intermittent running [68,127], but not short-duration HIIT [128,129,130]. However, one study showed a benefit of pre-exercise CHO ingestion on an exercise capacity test lasting ~8–10 min [67]. Several studies have compared high-fat and high-CHO pre-exercise meals with minimal performance differences observed [57,60,125,131].

#### 2.3.1. Amount, Type, and Timing of the Pre-Exercise Meal

The amount of CHO (25–312 g) consumed prior to exercise does not have a meaningful influence on time trial performance [5,52,53,55], while the glycemic index appears to have only a small impact that is more likely to be observed in time-to-exhaustion, but not time-trial performance tests [132]. No differences in performance have been observed following pre-exercise ingestion of solid vs. liquid CHO [43], solid vs. gel-based CHO [133,134], or fast-food vs. sport supplements [135]. Timing of the pre-exercise meal has minimal effects when consumed 15, 45, or 75 min [61], 15 or 60 min [129], or 5 or 35 min [58] before exercise, but CHO ingested 30 min before exercise resulted in better performance than 120 min before exercise [67]. Taken together, performing fed vs. fasted exercise appears to have a far larger effect on exercise performance than the amount or timing of the meals, unless the difference in meal timing is at least 90 min. There is some fear of hypoglycemia from consuming CHO between 30–60 min prior to exercise; however, despite occurring in a small number of cases, there does not appear to be any detrimental performance effects or any relationship between low blood glucose concentrations and performance [136].

#### 2.3.2. Athlete Perceptions and Behavior

The perception of breakfast is also a consideration when comparing the acute performance effects of pre-exercise CHO intake and fasted exercise. Trained cyclists completed a ~20 min cycling time-trial more quickly when they perceived that they had consumed breakfast (CHO or placebo) prior to the start of the exercise, compared with a fasted exercise session [137], and there was a 4% improvement in ~1 h time-trial performance when cyclists were told the placebo drink actually contained CHO compared with a blinded trial [138]. However, when a time-trial was preceded by 2 h of steady-state cycling, there were no placebo effects observed [139], suggesting placebo effects may be minimized with longer exercise durations. When undertaking exercise with reduced muscle glycogen levels, the perception of CHO availability augmented HIIT capacity, although performance was not restored to that of CHO consumption [140]. In a survey of endurance athletes, 26% agreed and 51% disagreed with the statement, “the quality of my workout is the same whether I eat or do not eat beforehand” [15], making it likely that a large inter-individual variation exists with regard to the perception of breakfast and its influence on performance.

#### 2.3.3. Summary and Future Directions

Overall, the importance of consuming CHO before exercise increases as the exercise duration increases and exercising in the fed vs. fasted state appears to have a far greater effect on performance than the size or timing of the meals. To better understand the influence of pre-exercise energy availability vs. CHO availability and its effects on HIIT, future studies should compare fed vs. fasted exercise, along with pre-exercise protein ingestion, in the absence of CHO, prior to both HIIT and steady-state performance tests.

## 3. Training Adaptations

The majority of research looking at pre-exercise nutrition interventions has been in relation to a single exercise session, with far fewer studies looking at the impact on training adaptations. This is relevant because acute responses to exercise do not always correspond with long-term adaptations. For example, increased fat oxidation observed when training with low vs. high CHO availability does not translate into longer-term increases in fat-burning capacity [141,142]. Likewise, blunting key mitochondrial signaling proteins with CHO ingestion during acute exercise did not impair training-induced improvements in performance or mitochondrial biogenesis [81,94]. Therefore, it is important to understand the changes that occur with chronic training rather than an acute bout of exercise alone.

### 3.1. Skeletal Muscle Adaptation

**Figure 8:**
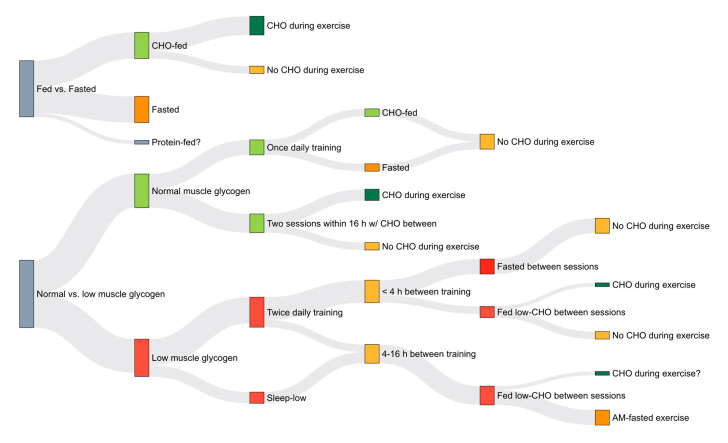
Comparison of the various methods of altering CHO availability used in training studies. Protocols used to commence training with a reduced availability of endogenous carbohydrate include overnight fasting, and training twice within a 24 h period consuming low-CHO nutrition between sessions or remaining in the fasted state. Some studies have fed carbohydrate during exercise, while others have not. Thickness of the line is related to the number of studies using a given approach. Question marks represent areas yet to be studied. Created from [12,13,14,31,35,93,141,142,143,144,145,148,149,150,151,152,153], which included 307 participants (10.7% female), 26.3 ± 4.2 years, VO_2max_ 53.2 ± 11.0 mL kg^−1^ min^−1^.

Of the studies examining the effects of longer-term (>4 weeks) training in the fasted state on endurance adaptations [12,13,14,143,144,145], only one [144] has used endurance-trained subjects. Furthermore, almost all studies using moderate-intensity continuous endurance training in the fasted state also provided the fed groups with CHO during exercise, which can independently influence both acute [120] and chronic [93] responses to exercise. Other studies have examined pre-exercise CHO supplementation, though not necessarily in the overnight-fasted state and using untrained subjects [146,147]. Additionally, fasted state training has been used as part of studies comparing low vs. high muscle glycogen [148] and once vs. twice daily training [149]. Therefore, making comparisons across studies is challenged by the variety of methods that have been used to compare high vs. low CHO availability around training sessions (Figure 8).

#### 3.1.1. Substrate Usage

One of the reasons athletes perform training sessions in the fasted state is a desire to increase fat oxidation during exercise [15]. As discussed above (Section 2.1), fat oxidation is higher during an acute bout of exercise performed in the overnight-fasted, compared with the CHO-fed state, and with low compared with high muscle glycogen. Despite these differences, most studies have found no differences in fat oxidation following 4–6 weeks of fed or fasted-state training when tested in the fed [13,14,145] or fasted [31,146] state. Similar findings have been reported in the “sleep-low” context, where fat oxidation is increased during fasted training sessions performed with low muscle glycogen compared with exercising in the fed-state, but no differences in fat oxidation were observed following one [148], three [142], or four [141] weeks of training when tested in the fed state. However, it is possible that longer time periods of fasted training may be needed before relevant differences in fat oxidation would be observed, as proteins involved in fat oxidation have been increased following fasted, but not fed-state training [12,14]. Studies that have reported improvements in fat oxidation following training with low compared with normal muscle glycogen tested subjects in the fasted state and trained twice-daily with only water ingested between the sessions [149,150]. Though speculative, these differences could be related to FFA signaling, which are increased during exercise and increased even further if no food is ingested in the hours following exercise [105]. Finally, IMTG usage during exercise was increased after 6 weeks of fasted (but not fed) training when tested in the fasted state [145], but there were no differences when tested in a fed state, while also providing additional CHO [14]. Taken together, it appears that increases in fat oxidation following fasted-state or low-glycogen training may not be relevant during typical racing conditions when consuming CHO before and during exercise, but more studies in endurance-trained athletes are needed to compare acute and chronic changes.

#### 3.1.2. Mitochondrial Markers

A key feature of the adaptive response to endurance training are changes in the activity of enzymes involved in the tricarboxylic acid (TCA) cycle and the β-oxidative pathway [154]. Activity of citrate synthase (CS) is the most widely used biomarker of mitochondrial content in skeletal muscle because of the strong correlation between resting CS activity and resting mitochondrial content when measured using the “gold standard” transmission electron microscopy (TEM) [155]. Similar changes in CS activity have been observed between fasted and fed-state training following 4–6 weeks of moderate-intensity training [12,13] and HIIT [143,146]. A key enzyme of the β-oxidative pathway, β-hydroxyacyl coenzyme A dehydrogenase (β-HAD), is also generally not impacted by pre-exercise nutrition [12,13,143]. However, one study has shown an increase in both CS and β-HAD only with fasted, but not CHO-fed training [145]. It is possible that this difference may be related to the very large amount of CHO ingested in the fed-training group (~2 g kg^−1^ 90 min prior and 1 g kg^−1^ h^−1^ during exercise), as other studies showing similar adaptations between fed and fasted training used smaller (e.g., 1–1.5 g kg^−1^ CHO) pre-training meals [13,143]. Increases in succinate dehydrogenase activity following twice-daily training were blunted when ingesting CHO before and during the second workout, which was commenced with lowered muscle glycogen [93], suggesting a strong, and potentially underappreciated influence of ingesting CHO during exercise that adds complexity when interpreting the current literature.

Greater increases in CS have been reported in two studies that had subjects train twice-daily every other day, inducing low muscle glycogen during the second bout of exercise, compared with once-daily training with normal muscle glycogen [35,150]. In these studies, the two sessions were 1–2 h apart and subjects received only water between sessions. In contrast, other studies using twice-daily training but feeding low- or high-CHO meals between sessions found similar training-induced increases in CS activity between groups [151,153]. When comparing two different “train-low” protocols (2 h vs. 15 h between low-glycogen training sessions), greater elevations in acute signaling and mitochondrial adaptations were observed when training with 2 h between sessions without ingesting any food [152,156]. Thus, it appears that remaining in the fasted state following the first bout of exercise may be an important factor in the augmented adaptations observed following twice-daily training.

Overall, the exercise training itself seems to be the primary driver of changes in mitochondrial content, though very large pre-exercise meals (>1.5 g/kg CHO) and CHO ingestion during exercise may have blunting effects on some signaling pathways, possibly related to the interactions between AMPK and glycogen [79]. Future research should explore the effects of pre-exercise nutrition choices on contraction-induced and RONS signaling pathways.

#### 3.1.3. VO_2max_ and Peak Aerobic Power

Studies comparing fasted and fed training have reported no differences in VO_2max_ following 4 weeks of sprint interval training (SIT) [144], 6 weeks of aerobic training [14,145], and 3 weeks of mixed intensity training [157]. However, greater training-induced increases in VO_2max_ have also been reported following both fasted vs. fed-state training [13] and fed vs. fasted-state training [12]. Reasons for these divergent findings are unclear, as both studies used untrained participants performing 4–6 weeks of steady-state aerobic training. Similar improvements in VO_2max_ and peak power were seen in untrained men following 8 weeks of HIIT with or without prior CHO [146], and following exercise undertaken with low or high muscle glycogen levels in trained and untrained athletes [35,93,153,158,159].

#### 3.1.4. Summary and Future Directions

Pre-exercise nutrition intake would not be expected to have an effect on VO_2max_ (which is largely affected by central adaptations [160]), but may affect peripheral adaptations that are influenced by fuel availability such as the substrate usage and mitochondrial size, particularly in untrained participants. Although there is some potential for pre-exercise nutrition intake to influence adaptations to endurance training, the lack of research in endurance-trained subjects, the very large amounts of CHO ingested before exercise in some studies, and the provision of CHO both before and during exercise in other studies makes extrapolating results to trained athletes challenging. Additionally, some of the strongest evidence suggesting low-glycogen training can magnify signaling responses to exercise is based on studies performing the experimental exercise session a few hours after a glycogen-lowering exercise bout [149,150,151], and some of these effects might simply be attributable to performing two exercise sessions in close proximity [156].

Future training studies should compare fasted-state training against low-CHO and moderate-CHO pre-exercise meals, with both normal and low muscle glycogen, and in the context of both HIIT and steady-state continuous endurance training to determine if there are differential effects on fat oxidation and/or mitochondrial biogenesis. It would also be of interest to investigate if there is a threshold for the amount of pre-exercise CHO ingested, independent of muscle glycogen levels [161], above which adaptations may be negatively impacted but below which adaptations are not impaired. Additionally, sex-based differences in the response to training programs should be investigated, as females accounted for just ~10% of participants in the training studies discussed.

### 3.2. Performance Changes

Studies comparing fed vs. fasted training have reported similar improvements in time-to-exhaustion during a maximal incremental test [145,162] and 1-h time-trial performance [145] following 6 weeks of endurance training. In contrast, time-to-fatigue at 85% VO_2max_ improved more in trained cyclists performing SIT in the fasted state compared to those that consumed CHO (>2.5 g kg^−1^ CHO prior and CHO drink during exercise), despite performing less work during training sessions [144]. Trained endurance athletes had greater improvements in a 12 min running time-trial following 3 weeks of aerobic training while consuming a low-GI compared with moderate GI diet [163].

Some studies comparing high vs. low glycogen training have reported similar performance improvements between groups [93,141,149,150,153], however greater improvements were seen following one and three weeks of sleep-low training [142,148], twice-daily training with low-CHO vs. high-CHO consumption between sessions [151], and twice- vs. once-daily training [35]. Two studies using a combination of tactics to vary CHO availability around training sessions (i.e., periodized-CHO) found similar improvements between chronic high-CHO and periodized-CHO diets, both of which resulted in greater improvements than a chronic low-CHO diet [158,159]. Future training studies should compare pre-exercise protein ingestion against CHO-fed and fasted-state training in the context of both HIIT and steady-state continuous endurance training. Additionally, it would be of interest to study whether a delayed CHO ingestion strategy [164] in the context of low glycogen or fasted-state training has any influence on the adaptive response and whether it is training specific (e.g., high- vs. low-intensity training).

## 4. Science to Practice

**Figure 9:**
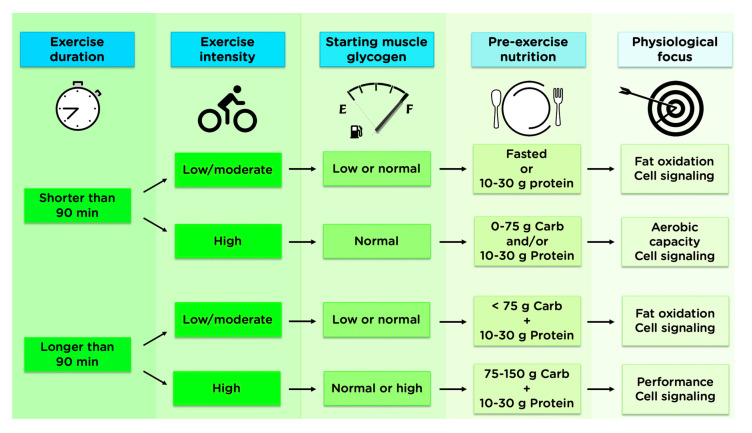
Practical application of pre-exercise nutrition to optimize training adaptations. The duration and intensity of the exercise session should be considered when considering the best pre-exercise nutrition choices. Before shorter duration exercise sessions that focus on lower intensity steady-state training, it may be beneficial to withhold CHO, while there is little evidence supporting CHO restriction before high-intensity exercise. When consuming less than ~75 g CHO, food choices before HIIT can be left to personal preference. For longer duration exercise (>90 min), there is little evidence to suggest fasted-state training offers any additional benefit, although this is still practiced by approximately one-third of endurance athletes [16]. Ingesting less than ~75 g CHO is unlikely to impair mitochondrial signaling adaptations from longer-duration, low-intensity exercise, while consuming 75–150 g CHO prior to extended high-intensity exercise is suggested to increase endogenous fuel storage.

In an attempt to optimize both training adaptations and acute performance during key training sessions, current sport nutrition guidelines suggest training be performed both with high CHO availability, in order to enhance glycolytic and CHO oxidation pathways, and low CHO availability to increase the activation of acute cell signaling pathways related to mitochondrial biogenesis and fat oxidation [3]. Despite the rationale for a periodized approach to nutrition, whereby CHO availability for each workout is varied according to the type of session and its goals within a periodized training cycle [161], many athletes are not following these recommendations and/or are unclear on the current best-practice guidelines. For example, only 17–27% of elite athletes report following a periodized-CHO diet, and less than half of endurance athletes report varying their pre-exercise nutrition choices based on exercise duration or intensity [16,165]. Although training in the overnight-fasted state is performed by nearly two-thirds of endurance athletes (63%), many are doing it because they think it is beneficial, while others avoid it because they think it is not beneficial [15]. Furthermore, nearly all beliefs and practices relating to pre-exercise nutrition appear to vary based on sex, competitive level, and habitual dietary pattern [15,16]. Taken together, this highlights the need for more research in trained athletes as well as improved communication of the available research to athletes and coaches. From the standpoint of practical application, the duration and intensity of the exercise session should be considered when considering the best pre-exercise nutrition choices, along with the personal preferences of each individual athlete, as described in Figure 9. While the principles behind these recommendations should be applicable to a broad population, the relative influence of nutrition on training adaptations may vary based on sex, BMI, and training status.

## 5. Conclusions and Practical Application

The availability of endogenous and exogenous CHO, fat, and protein before and during exercise can influence the acute and longer-term responses to endurance exercise. Acutely, CHO ingestion inhibits fat burning, however evidence showing enhanced fat burning capacity following long-term training in the fasted state is lacking. Contrasting findings related to the influence of CHO ingestion on mitochondrial signaling may be related to the amount of carbohydrate consumed and the intensity of exercise. Consumption of >120 g CHO before submaximal, steady-state exercise has blunted mitochondrial signaling, while <70 g CHO has not, yet CHO availability appears to have minimal effects following HIIT exercise. Performance is improved following pre-exercise CHO ingestion for longer but not shorter duration exercise, while training-induced performance changes following various pre-exercise nutrition strategies vary based on the type of nutrition protocol used. Caution should be used when generalizing these findings to wider populations, as the majority of research participants have been trained males between 20–30 years of age. In addition to wider participant demographics, more research is needed on the acute and longer-term effects of pre-exercise protein ingestion, studies in endurance-trained subjects performing fasted-state training compared with ingesting moderate and low-CHO meals before exercise, and fasted vs. fed-state training without CHO ingestion during exercise.

## Supplementary Materials

The following are available online at https://www.mdpi.com/2072-6643/12/11/3473/s1, References for studies included in pooled analyses.
