# Supplementary material for: What Should I Eat before Exercise? Pre-Exercise Nutrition and the Response to Endurance Exercise: Current Prospective and Future Directions
Source: Nutrients. 2020 Nov 12;12(11):3473. doi: 10.3390/nu12113473 (PMC7696145; doi:10.3390/nu12113473)
Supplement: Supplementary file 1 [file nutrients-12-03473-s001.pdf]

References for studies used in pooled analyses

Figure 2. Substrate oxidation in relation to exercise time for studies reporting RER at multiple time points.

Data were pooled from [1–45] for Fig. 2 a (CHO-fed vs. fasted), from [27,29,34,46–55] for Fig 2.b (glycogen), and from[2–4,23,30,56–63] for Fig. 2 c. (glycemic index).

Figure 3. Correlation between differences in respiratory exchange ratio (RER) during exercise and differences in pre-exercise glycogen levels.

Data were obtained by pooling results from [27,29,34,46,48,49,51,64–69].

Figure 4. Substrate oxidation in relation to exercise intensity

Data were pooled from [1–26,28–32,35–40,42,43,55,70–102] for Fig. 4a,b, from [27,29,34,46–54,64–69,103–105] for Fig. 4c,d, and from [2–4,23,30,56–62,72,75,84,86,97,106–108] for Fig. 4e,f.

Figure 5. Substrate oxidation in relation to amount of CHO consumed before exercise.

Data pooled from [1–40,42,43,70–102,109].

Figure 6. Substrate oxidation in relation to the time food was consumed before exercise.

Data pooled from [1–40,42,43,70–102,109].

Figure 7. Effects of nutrition related factors on AMPKa2 activity during exercise.

Data pooled from [27,29,44,48,51,88,89,110–125].

1. Chryssanthopoulos, C.; Hennessy, L.C.; Williams, C. The influence of pre-exercise glucose ingestion on endurance running capacity. *Br J Sports Med* **1994**, *28*, 105–109, doi:10.1136/bjsm.28.2.105.
2. DeMarco, H.M.; Sucher, K.P.; Cisar, C.J.; Butterfield, G.E. Pre-exercise carbohydrate meals: application of glycemic index. *Med Sci Sports Exerc* **1999**, *31*, 164–170.
3. Jamurtas, A.Z.; Tofas, T.; Fatouros, I.; Nikolaidis, M.G.; Paschalis, V.; Yfanti, C.; Raptis, S.; Koutedakis, Y. The effects of low and high glycemic index foods on exercise performance and beta-endorphin responses. *J Int Soc Sports Nutr* **2011**, *8*, 15, doi:10.1186/1550-2783-8-15.
4. Kirwan, J.P.; Cyr-Campbell, D.; Campbell, W.W.; Scheiber, J.; Evans, W.J. Effects of moderate and high glycemic index meals on metabolism and exercise performance. *Metabolism* **2001**, *50*, 849–855, doi:10.1053/meta.2001.24191.
5. Kirwan, J.P.; O'Gorman, D.; Evans, W.J. A moderate glycemic meal before endurance exercise can enhance performance. *J Appl Physiol* **1998**, *84*, 53–59.
6. Neufer, P.D.; Costill, D.L.; Flynn, M.G.; Kirwan, J.P.; Mitchell, J.B.; Houmard, J. Improvements in exercise performance: effects of carbohydrate feedings and diet. *J Appl Physiol (1985)* **1987**, *62*, 983–988, doi:10.1152/jappl.1987.62.3.983.
7. Schabort, E.J.; Bosch, A.N.; Weltan, S.M.; Noakes, T.D. The effect of a preexercise meal on time to fatigue during prolonged cycling exercise. *Med Sci Sports Exerc* **1999**, *31*, 464–471.
8. Stocks, B.; Dent, J.R.; Ogden, H.B.; Zemp, M.; Philp, A. Postexercise skeletal muscle signaling responses to moderate- to high-intensity steady-state exercise in the fed or fasted state. *Am J Physiol Endocrinol Metab* **2019**, *316*, E230–E238, doi:10.1152/ajpendo.00311.2018.
9. Chryssanthopoulos, C.; Williams, C.; Nowitz, A.; Kotsiopoulou, C.; Vleck, V. The effect of a high carbohydrate meal on endurance running capacity. *Int J Sport Nutr Exerc Metab* **2002**, *12*, 157–171.
10. Tokmakidis, S.P.; Karamanolis, I.A. Effects of carbohydrate ingestion 15 min before exercise on endurance running capacity. *Appl Physiol Nutr Metab* **2008**, *33*, 441–449.

11. Flynn, M.G.; Michaud, T.J.; Rodriguez-Zayas, J.; Lambert, C.P.; Boone, J.B.; Moleski, R.W. Effects of 4-and 8-h preexercise feedings on substrate use and performance. *J Appl Physiol* **1989**, *67*, 2066–2071.
12. Bergman, B.C.; Brooks, G.A. Respiratory gas-exchange ratios during graded exercise in fed and fasted trained and untrained men. *J Appl Physiol (1985)* **1999**, *86*, 479–487, doi:10.1152/jappl.1999.86.2.479.
13. Coyle, E.F.; Coggan, A.R.; Hemmert, M.K.; Lowe, R.C.; Walters, T.J. Substrate usage during prolonged exercise following a preexercise meal. *J Appl Physiol (1985)* **1985**, *59*, 429–433, doi:10.1152/jappl.1985.59.2.429.
14. Cramp, T.; Broad, E.; Martin, D.; Meyer, B.J. Effects of preexercise carbohydrate ingestion on mountain bike performance. *Med Sci Sports Exerc* **2004**, *36*, 1602–1609.
15. Gleeson, M.; Maughan, R.J.; Greenhaff, P.L. Comparison of the effects of pre-exercise feeding of glucose, glycerol and placebo on endurance and fuel homeostasis in man. *Eur J Appl Physiol Occup Physiol* **1986**, *55*, 645–653.
16. Isacco, L.; Thivel, D.; Pelle, A.M.; Zouhal, H.; Duclos, M.; Duche, P.; Boisseau, N. Oral contraception and energy intake in women: impact on substrate oxidation during exercise. *Appl Physiol Nutr Metab* **2012**, *37*, 646–656, doi:10.1139/h2012-031.
17. Learsi, S.K.; Ghiarone, T.; Silva-Cavalcante, M.D.; Andrade-Souza, V.A.; Ataide-Silva, T.; Bertuzzi, R.; de Araujo, G.G.; McConell, G.; Lima-Silva, A.E. Cycling time trial performance is improved by carbohydrate ingestion during exercise regardless of a fed or fasted state. *Scand J Med Sci Sports* **2019**.
18. Murakami, I.; Sakuragi, T.; Uemura, H.; Menda, H.; Shindo, M.; Tanaka, H. Significant effect of a pre-exercise high-fat meal after a 3-day high-carbohydrate diet on endurance performance. *Nutrients* **2012**, *4*, 625–637, doi:10.3390/nu4070625.
19. Okano, G.; Sato, Y.; Takumi, Y.; Sugawara, M. Effect of 4h preexercise high carbohydrate and high fat meal ingestion on endurance performance and metabolism. *Int J Sports Med* **1996**, *17*, 530–534, doi:10.1055/s-2007-972890.
20. Paul, G.L.; Rokusek, J.T.; Dykstra, G.L.; Boileau, R.A.; Layman, D.K. Oat, wheat or corn cereal ingestion before exercise alters metabolism in humans. *J Nutr* **1996**, *126*, 1372–1381.
21. Satabin, P.; Portero, P.; Defer, G.; Bricout, J.; Guezennec, C.Y. Metabolic and hormonal responses to lipid and carbohydrate diets during exercise in man. *Med Sci Sports Exerc* **1987**, *19*, 218–223.
22. Sherman, W.M.; Brodowicz, G.; Wright, D.A.; Allen, W.K.; Simonsen, J.; Dernbach, A. Effects of 4 h preexercise carbohydrate feedings on cycling performance. *Med Sci Sports Exerc* **1989**, *21*, 598–604.
23. Thomas, D.; Brotherhood, J.; Brand, J. Carbohydrate feeding before exercise: effect of glycemic index. *Int J Sports Med* **1991**, *12*, 180–186.
24. van Loon, L.J.; Koopman, R.; Stegen, J.H.; Wagenmakers, A.J.; Keizer, H.A.; Saris, W.H. Intramyocellular lipids form an important substrate source during moderate intensity exercise in endurance-trained males in a fasted state. *J Physiol* **2003**, *553*, 611–625, doi:10.1113/jphysiol.2003.052431.
25. Whitley, H.A.; Humphreys, S.M.; Campbell, I.T.; Keegan, M.A.; Jayanetti, T.D.; Sperry, D.A.; MacLaren, D.P.; Reilly, T.; Frayn, K.N. Metabolic and performance responses during endurance exercise after high-fat and high-carbohydrate meals. *J Appl Physiol (1985)* **1998**, *85*, 418–424, doi:10.1152/jappl.1998.85.2.418.
26. Wright, D.; Sherman, W.; Dernbach, A. Carbohydrate feedings before, during, or in combination improve cycling endurance performance. *J Appl Physiol* **1991**, *71*, 1082–1088.
27. Wojtaszewski, J.F.; MacDonald, C.; Nielsen, J.N.; Hellsten, Y.; Hardie, D.G.; Kemp, B.E.; Kiens, B.; Richter, E.A. Regulation of 5′ AMP-activated protein kinase activity and substrate utilization in exercising human skeletal muscle. *Am J Physiol Endocrinol Metab* **2003**, *284*, E813–E822.
28. Costill, D.L.; Coyle, E.; Dalsky, G.; Evans, W.; Fink, W.; Hoopes, D. Effects of elevated plasma FFA and insulin on muscle glycogen usage during exercise. *J Appl Physiol Respir Environ Exerc Physiol* **1977**, *43*, 695–699, doi:10.1152/jappl.1977.43.4.695.
29. Steinberg, G.R.; Watt, M.J.; McGee, S.L.; Chan, S.; Hargreaves, M.; Febbraio, M.A.; Stapleton, D.; Kemp, B.E. Reduced glycogen availability is associated with increased AMPKα2 activity, nuclear AMPKα2 protein abundance, and GLUT4 mRNA expression in contracting human skeletal muscle. *Appl Physiol Nutr Metab* **2006**, *31*, 302–312.
30. Backhouse, S.H.; Williams, C.; Stevenson, E.; Nute, M. Effects of the glycemic index of breakfast on metabolic responses to brisk walking in females. *Eur J Clin Nutr* **2007**, *61*, 590–596, doi:10.1038/sj.ejcn.1602566.

31. el-Sayed, M.S.; Balmer, J.; Rattu, A.J. Carbohydrate ingestion improves endurance performance during a 1 h simulated cycling time trial. *J Sports Sci* **1997**, *15*, 223–230, doi:10.1080/026404197367506.
32. Coggan, A.R.; Coyle, E.F. Metabolism and performance following carbohydrate ingestion late in exercise. *Med Sci Sports Exerc* **1989**, *21*, 59–65, doi:10.1249/00005768-198902000-00011.
33. Arkinstall, M.J.; Bruce, C.R.; Nikolopoulos, V.; Garnham, A.P.; Hawley, J.A. Effect of carbohydrate ingestion on metabolism during running and cycling. *J Appl Physiol (1985)* **2001**, *91*, 2125–2134, doi:10.1152/jappl.2001.91.5.2125.
34. Arkinstall, M.J.; Bruce, C.R.; Clark, S.A.; Rickards, C.A.; Burke, L.M.; Hawley, J.A. Regulation of fuel metabolism by preexercise muscle glycogen content and exercise intensity. *J Appl Physiol (1985)* **2004**, *97*, 2275–2283, doi:10.1152/japplphysiol.00421.2004.
35. Coggan, A.R.; Raguso, C.A.; Gastaldelli, A.; Sidossis, L.S.; Yeckel, C.W. Fat metabolism during high-intensity exercise in endurance-trained and untrained men. *Metabolism* **2000**, *49*, 122–128, doi:10.1016/s0026-0495(00)90963-6.
36. Coyle, E.F.; Jeukendrup, A.E.; Wagenmakers, A.; Saris, W. Fatty acid oxidation is directly regulated by carbohydrate metabolism during exercise. *American Journal of Physiology-Endocrinology And Metabolism* **1997**, *273*, E268–E275.
37. Tarnopolsky, L.J.; MacDougall, J.D.; Atkinson, S.A.; Tarnopolsky, M.A.; Sutton, J.R. Gender differences in substrate for endurance exercise. *J Appl Physiol (1985)* **1990**, *68*, 302–308, doi:10.1152/jappl.1990.68.1.302.
38. Phillips, S.M.; Atkinson, S.A.; Tarnopolsky, M.A.; MacDougall, J.D. Gender differences in leucine kinetics and nitrogen balance in endurance athletes. *J Appl Physiol (1985)* **1993**, *75*, 2134–2141, doi:10.1152/jappl.1993.75.5.2134.
39. Costill, D.L.; Fink, W.J.; Getchell, L.H.; Ivy, J.L.; Witzmann, F.A. Lipid metabolism in skeletal muscle of endurance-trained males and females. *J Appl Physiol Respir Environ Exerc Physiol* **1979**, *47*, 787–791, doi:10.1152/jappl.1979.47.4.787.
40. Foster, C.; Costill, D.; Fink, W. Effects of preexercise feedings on endurance performance. *Med Sci Sports* **1979**, *11*, 1–5.
41. Watt, M.J.; Heigenhauser, G.J.; Dyck, D.J.; Spriet, L.L. Intramuscular triacylglycerol, glycogen and acetyl group metabolism during 4 h of moderate exercise in man. *J Physiol* **2002**, *541*, 969–978, doi:10.1113/jphysiol.2002.018820.
42. Shin, Y.H.; Jung, H.L.; Ryu, J.W.; Kim, P.S.; Ha, T.Y.; An, J.Y.; Kang, H.Y. Effects of a Pre-Exercise Meal on Plasma Growth Hormone Response and Fat Oxidation during Walking. *Prev Nutr Food Sci* **2013**, *18*, 175–180, doi:10.3746/pnf.2013.18.3.175.
43. Van Proeyen, K.; Szlufcik, K.; Nielens, H.; Ramaekers, M.; Hespel, P. Beneficial metabolic adaptations due to endurance exercise training in the fasted state. *J Appl Physiol (1985)* **2011**, *110*, 236–245.
44. Wojtaszewski, J.F.; Mourtzakis, M.; Hillig, T.; Saltin, B.; Pilegaard, H. Dissociation of AMPK activity and ACCbeta phosphorylation in human muscle during prolonged exercise. *Biochem Biophys Res Commun* **2002**, *298*, 309–316, doi:10.1016/s0006-291x(02)02465-8.
45. van Loon, L.J.; Greenhaff, P.L.; Constantin-Teodosiu, D.; Saris, W.H.; Wagenmakers, A.J. The effects of increasing exercise intensity on muscle fuel utilisation in humans. *J Physiol* **2001**, *536*, 295–304.
46. Alghannam, A.F.; Jedrzejewski, D.; Tweddle, M.G.; Gribble, H.; Bilzon, J.; Thompson, D.; Tsintzas, K.; Betts, J.A. Impact of Muscle Glycogen Availability on the Capacity for Repeated Exercise in Man. *Med Sci Sports Exerc* **2016**, *48*, 123–131, doi:10.1249/MSS.0000000000000737.
47. Impey, S.G.; Smith, D.; Robinson, A.L.; Owens, D.J.; Bartlett, J.D.; Smith, K.; Limb, M.; Tang, J.; Fraser, W.D.; Close, G.L. Leucine-enriched protein feeding does not impair exercise-induced free fatty acid availability and lipid oxidation: beneficial implications for training in carbohydrate-restricted states. *Amino acids* **2015**, *47*, 407–416.
48. Lane, S.C.; Camera, D.M.; Lassiter, D.G.; Areta, J.L.; Bird, S.R.; Yeo, W.K.; Jeacocke, N.A.; Krook, A.; Zierath, J.R.; Burke, L.M., et al. Effects of sleeping with reduced carbohydrate availability on acute training responses. *J Appl Physiol (1985)* **2015**, *119*, 643–655, doi:10.1152/japplphysiol.00857.2014.
49. Margolis, L.M.; Wilson, M.A.; Whitney, C.C.; Carrigan, C.T.; Murphy, N.E.; Hatch, A.M.; Montain, S.J.; Pasiakos, S.M. Exercising with low muscle glycogen content increases fat oxidation and decreases endogenous, but not exogenous carbohydrate oxidation. *Metabolism* **2019**, *97*, 1–8.

50. Pilegaard, H.; Keller, C.; Steensberg, A.; Helge, J.W.; Pedersen, B.K.; Saltin, B.; Neufer, P.D. Influence of pre-exercise muscle glycogen content on exercise-induced transcriptional regulation of metabolic genes. *J Physiol* **2002**, *541*, 261–271, doi:10.1113/jphysiol.2002.016832.
51. Roepstorff, C.; Halberg, N.; Hillig, T.; Saha, A.K.; Ruderman, N.B.; Wojtaszewski, J.F.; Richter, E.A.; Kiens, B. Malonyl-CoA and carnitine in regulation of fat oxidation in human skeletal muscle during exercise. *Am J Physiol Endocrinol Metab* **2005**, *288*, E133–E142, doi:10.1152/ajpendo.00379.2004.
52. Taylor, C.; Bartlett, J.D.; van de Graaf, C.S.; Louhelainen, J.; Coyne, V.; Iqbal, Z.; MacLaren, D.P.; Gregson, W.; Close, G.L.; Morton, J.P. Protein ingestion does not impair exercise-induced AMPK signalling when in a glycogen-depleted state: implications for train-low compete-high. *Eur J Appl Physiol* **2013**, *113*, 1457–1468.
53. Waterworth, S.P.; Spencer, C.C.; Porter, A.L.; Morton, J.P. Perception of Carbohydrate Availability Augments High-Intensity Intermittent Exercise Capacity Under Sleep-Low, Train-Low Conditions. *Int J Sport Nutr Exerc Metab* **2020**, 10.1123/ijsnem.2019-0275, 1-7, doi:10.1123/ijsnem.2019-0275.
54. Areta, J.L.; Iraki, J.; Owens, D.J.; Joanisse, S.; Philp, A.; Morton, J.P.; Hallén, J. Achieving energy balance with a high-fat meal does not enhance skeletal muscle adaptation and impairs glycemic response in a sleep-low training model. *Exp Physiol* **2020**.
55. Roepstorff, C.; Steffensen, C.H.; Madsen, M.; Stallknecht, B.; Kanstrup, I.L.; Richter, E.A.; Kiens, B. Gender differences in substrate utilization during submaximal exercise in endurance-trained subjects. *Am J Physiol Endocrinol Metab* **2002**, *282*, E435–E447, doi:10.1152/ajpendo.00266.2001.
56. Moore, L.J.; Midgley, A.W.; Thurlow, S.; Thomas, G.; Mc Naughton, L.R. Effect of the glycaemic index of a pre-exercise meal on metabolism and cycling time trial performance. *J Sci Med Sport* **2010**, *13*, 182–188, doi:10.1016/j.jsams.2008.11.006.
57. Moore, L.J.; Midgley, A.W.; Thomas, G.; Thurlow, S.; McNaughton, L.R. The effects of low- and high-glycemic index meals on time trial performance. *Int J Sports Physiol Perform* **2009**, *4*, 331–344, doi:10.1123/ijspp.4.3.331.
58. Wee, S.L.; Williams, C.; Gray, S.; Horabin, J. Influence of high and low glycemic index meals on endurance running capacity. *Med Sci Sports Exerc* **1999**, *31*, 393–399, doi:10.1097/00005768-199903000-00007.
59. Burke, L.M.; Claassen, A.; Hawley, J.A.; Noakes, T.D. Carbohydrate intake during prolonged cycling minimizes effect of glycemic index of preexercise meal. *J Appl Physiol (1985)* **1998**, *85*, 2220–2226, doi:10.1152/jappl.1998.85.6.2220.
60. Chen, Y.J.; Wong, S.H.; Wong, C.K.; Lam, C.W.; Huang, Y.J.; Siu, P.M. Effect of preexercise meals with different glycemic indices and loads on metabolic responses and endurance running. *Int J Sport Nutr Exerc Metab* **2008**, *18*, 281–300, doi:10.1123/ijsnem.18.3.281.
61. Roberts, M.D.; Lockwood, C.; Dalbo, V.J.; Volek, J.; Kerksick, C.M. Ingestion of a high-molecular-weight hydrothermally modified waxy maize starch alters metabolic responses to prolonged exercise in trained cyclists. *Nutrition* **2011**, *27*, 659–665, doi:10.1016/j.nut.2010.07.008.
62. Thomas, D.E.; Brotherhood, J.R.; Miller, J.B. Plasma glucose levels after prolonged strenuous exercise correlate inversely with glycemic response to food consumed before exercise. *Int J Sport Nutr Exerc Metab* **1994**, *4*, 361–373.
63. Gejl, K.D.; Vissing, K.; Hansen, M.; Thams, L.; Rokkedal-Lausch, T.; Plomgaard, P.; Meinild Lundby, A.K.; Nybo, L.; Jensen, K.; Holmberg, H.C. Changes in metabolism but not myocellular signaling by training with CHO-restriction in endurance athletes. *Physiol Rep* **2018**, *6*, e13847.
64. Bergstrom, J.; Hermansen, L.; Hultman, E.; Saltin, B. Diet, muscle glycogen and physical performance. *Acta Physiol Scand* **1967**, *71*, 140–150, doi:10.1111/j.1748-1716.1967.tb03720.x.
65. Hargreaves, M.; McConell, G.; Proietto, J. Influence of muscle glycogen on glycogenolysis and glucose uptake during exercise in humans. *J Appl Physiol (1985)* **1995**, *78*, 288–292, doi:10.1152/jappl.1995.78.1.288.
66. Hearris, M.A.; Owens, D.J.; Strauss, J.A.; Shepherd, S.O.; Sharples, A.P.; Morton, J.P.; Louis, J.B. Graded reductions in pre-exercise glycogen concentration do not augment exercise-induced nuclear AMPK and PGC-1α protein content in human muscle. *Exp Physiol* **2020**, *In Press*, doi:10.1113/ep088866.
67. Howarth, K.R.; Phillips, S.M.; MacDonald, M.J.; Richards, D.; Moreau, N.A.; Gibala, M.J. Effect of glycogen availability on human skeletal muscle protein turnover during exercise and recovery. *J Appl Physiol (1985)* **2010**, *109*, 431–438, doi:10.1152/japplphysiol.00108.2009.

68. Kirwan, J.P.; Costill, D.L.; Mitchell, J.B.; Houmard, J.A.; Flynn, M.G.; Fink, W.J.; Beltz, J.D. Carbohydrate balance in competitive runners during successive days of intense training. *J Appl Physiol (1985)* **1988**, *65*, 2601–2606, doi:10.1152/jappl.1988.65.6.2601.
69. Widrick, J.J.; Costill, D.L.; Fink, W.J.; Hickey, M.S.; McConell, G.K.; Tanaka, H. Carbohydrate feedings and exercise performance: effect of initial muscle glycogen concentration. *J Appl Physiol (1985)* **1993**, *74*, 2998–3005, doi:10.1152/jappl.1993.74.6.2998.
70. Alberici, J.C.; Farrell, P.A.; Kris-Etherton, P.M.; Shively, C.A. Effects of preexercise candy bar ingestion on glycemic response, substrate utilization, and performance. *Int J Sport Nutr* **1993**, *3*, 323–333.
71. Bachman, J.L.; Deitrick, R.W.; Hillman, A.R. Exercising in the Fasted State Reduced 24-Hour Energy Intake in Active Male Adults. *J Nutr Metab* **2016**, *2016*, 1984198, doi:10.1155/2016/1984198.
72. Bennard, P.; Doucet, E. Acute effects of exercise timing and breakfast meal glycemic index on exercise-induced fat oxidation. *Appl Physiol Nutr Metab* **2006**, *31*, 502–511, doi:10.1139/h06-027.
73. Enevoldsen, L.; Simonsen, L.; Macdonald, I.; Bülow, J. The combined effects of exercise and food intake on adipose tissue and splanchnic metabolism. *J. Physiol* **2004**, *561*, 871–882.
74. Febbraio, M.A.; Keenan, J.; Angus, D.J.; Campbell, S.E.; Garnham, A.P. Preexercise carbohydrate ingestion, glucose kinetics, and muscle glycogen use: effect of the glycemic index. *J. Appl Physiol* **2000**, *89*, 1845–1851.
75. Febbraio, M.A.; Stewart, K.L. CHO feeding before prolonged exercise: effect of glycemic index on muscle glycogenolysis and exercise performance. *J. Appl Physiol* **1996**, *81*, 1115–1120.
76. Gillen, J.B.; West, D.W.D.; Williamson, E.P.; Fung, H.J.W.; Moore, D.R. Low-Carbohydrate Training Increases Protein Requirements of Endurance Athletes. *Med. Sci Sports Exerc* **2019**, *51*, 2294–2301, doi:10.1249/MSS.0000000000002036.
77. Jentjens, R.L.; Cale, C.; Gutch, C.; Jeukendrup, A.E. Effects of pre-exercise ingestion of differing amounts of carbohydrate on subsequent metabolism and cycling performance. *Eur J. Appl Physiol* **2003**, *88*, 444–452, doi:10.1007/s00421-002-0727-9.
78. Kang, J.; Raines, E.; Rosenberg, J.; Ratamess, N.; Naclerio, F.; Faigenbaum, A. Metabolic responses during postprandial exercise. *Res. Sports Med.* **2013**, *21*, 240–252, doi:10.1080/15438627.2013.792088.
79. Marmy-Conus, N.; Fabris, S.; Proietto, J.; Hargreaves, M. Preexercise glucose ingestion and glucose kinetics during exercise. *J. Appl Physiol (1985)* **1996**, *81*, 853–857, doi:10.1152/jappl.1996.81.2.853.
80. Mcmurray, R.G.; Wilson, J.R.; Kitchell, B.S. The effects of fructose and glucose on high intensity endurance performance. *Research Quarterly for Exercise and sport* **1983**, *54*, 156–162.
81. Moseley, L.; Lancaster, G.I.; Jeukendrup, A.E. Effects of timing of pre-exercise ingestion of carbohydrate on subsequent metabolism and cycling performance. *Eur J. Appl Physiol* **2003**, *88*, 453–458, doi:10.1007/s00421-002-0728-8.
82. Rowlands, D.S.; Hopkins, W.G. Effect of high-fat, high-carbohydrate, and high-protein meals on metabolism and performance during endurance cycling. *Int J. Sport Nutr Exerc Metab* **2002**, *12*, 318–335.
83. Sherman, W.M.; Peden, M.C.; Wright, D.A. Carbohydrate feedings 1 h before exercise improves cycling performance. *Am. J. Clin. Nutr* **1991**, *54*, 866–870.
84. Sparks, M.J.; Selig, S.S.; Febbraio, M.A. Pre-exercise carbohydrate ingestion: effect of the glycemic index on endurance exercise performance. *Med. Sci Sports Exerc* **1998**, *30*, 844–849, doi:10.1097/00005768-199806000-00011.
85. Willcutts, K.F.; Wilcox, A.; Grunewald, K. Energy metabolism during exercise at different time intervals following a meal. *Int J. Sports Med.* **1988**, *9*, 240–243.
86. Wu, C.L.; Nicholas, C.; Williams, C.; Took, A.; Hardy, L. The influence of high-carbohydrate meals with different glycaemic indices on substrate utilisation during subsequent exercise. *Br. J. Nutr* **2003**, *90*, 1049–1056.
87. Egan, B.; Carson, B.P.; Garcia-Roves, P.M.; Chibalin, A.V.; Sarsfield, F.M.; Barron, N.; McCaffrey, N.; Moyna, N.M.; Zierath, J.R.; O'Gorman, D.J. Exercise intensity-dependent regulation of peroxisome proliferator-activated receptor γ coactivator-1α mRNA abundance is associated with differential activation of upstream signalling kinases in human skeletal muscle. *J. Physiol* **2010**, *588*, 1779–1790.
88. McConell, G.K.; Wadley, G.D.; Le Plastrier, K.; Linden, K.C. Skeletal muscle AMPK is not activated during 2 hours of moderate intensity exercise at~ 65% VO2 peak in endurance trained men. *J. Physiol* **2020**.

89. Yeo, W.K.; Lessard, S.J.; Chen, Z.-P.; Garnham, A.P.; Burke, L.M.; Rivas, D.A.; Kemp, B.E.; Hawley, J.A. Fat adaptation followed by carbohydrate restoration increases AMPK activity in skeletal muscle from trained humans. *J. Appl Physiol* **2008**, *105*, 1519–1526.
90. Calles-Escandón, J.; Devlin, J.T.; Whitcomb, W.; Horton, E.S. Pre-exercise feeding does not affect endurance cycle exercise but attenuates post-exercise starvation-like response. *Med. Sci Sports Exerc* **1991**, *23*, 818–824.
91. Edinburgh, R.M.; Hengist, A.; Smith, H.A.; Travers, R.L.; Betts, J.A.; Thompson, D.; Walhin, J.-P.; Wallis, G.A.; Hamilton, D.L.; Stevenson, E.J. Skipping Breakfast Before Exercise Creates a More Negative 24-hour Energy Balance: A Randomized Controlled Trial in Healthy Physically Active Young Men. *J. Nutr* **2019**.
92. Goodpaster, B.H.; Costill, D.L.; Fink, W.J.; Trappe, T.A.; Jozsi, A.C.; Starling, R.D.; Trappe, S.W. The effects of pre-exercise starch ingestion on endurance performance. *Int J. Sports Med.* **1996**, *17*, 366–372, doi:10.1055/s-2007-972862.
93. Massicotte, D.; Peronnet, F.; Brisson, G.; Boivin, L.; Hillaire-Marcel, C. Oxidation of exogenous carbohydrate during prolonged exercise in fed and fasted conditions. *Int J. Sports Med.* **1990**, *11*, 253–258, doi:10.1055/s-2007-1024802.
94. Romijn, J.A.; Coyle, E.F.; Sidossis, L.S.; Gastaldelli, A.; Horowitz, J.F.; Endert, E.; Wolfe, R.R. Regulation of endogenous fat and carbohydrate metabolism in relation to exercise intensity and duration. *Am. J. Physiol* **1993**, *265*, E380–E391, doi:10.1152/ajpendo.1993.265.3.E380.
95. Rollo, I.; Williams, C. Influence of ingesting a carbohydrate-electrolyte solution before and during a 1-hr running performance test. *Int J. Sport Nutr Exerc Metab* **2009**, *19*, 645–658.
96. Hamzah, S.; Higgins, S.; Abraham, T.; Taylor, P.; Vizbaraite, D.; Malkova, D. The effect of glycaemic index of high carbohydrate diets consumed over 5 days on exercise energy metabolism and running capacity in males. *J. Sports Sci* **2009**, *27*, 1545–1554, doi:10.1080/02640410903134115.
97. Baur, D.A.; Willingham, B.D.; Smith, K.A.; Kisiolek, J.N.; Morrissey, M.C.; Saracino, P.G.; Ragland, T.J.; Ormsbee, M.J. Adipose Lipolysis Unchanged by Preexercise Carbohydrate Regardless of Glycemic Index. *Med. Sci Sports Exerc* **2018**, *50*, 827–836, doi:10.1249/MSS.0000000000001498.
98. Horton, T.J.; Pagliassotti, M.J.; Hobbs, K.; Hill, J.O. Fuel metabolism in men and women during and after long-duration exercise. *J. Appl Physiol (1985)* **1998**, *85*, 1823–1832, doi:10.1152/jappl.1998.85.5.1823.
99. Romijn, J.A.; Coyle, E.F.; Sidossis, L.S.; Rosenblatt, J.; Wolfe, R.R. Substrate metabolism during different exercise intensities in endurance-trained women. *J. Appl Physiol (1985)* **2000**, *88*, 1707–1714, doi:10.1152/jappl.2000.88.5.1707.
100. Ruby, B.C.; Coggan, A.R.; Zderic, T.W. Gender differences in glucose kinetics and substrate oxidation during exercise near the lactate threshold. *J. Appl Physiol (1985)* **2002**, *92*, 1125–1132, doi:10.1152/japplphysiol.00296.2001.
101. Ziogas, G.; Thomas, T.R. Dietary preparation before rest and exercise testing. *Nutrition* **1998**, *14*, 11–16, doi:10.1016/s0899-9007(97)00387-0.
102. Edinburgh, R.M.; Bradley, H.E.; Abdullah, N.-F.; Robinson, S.L.; Chrzanowski-Smith, O.J.; Walhin, J.-P.; Joanisse, S.; Manolopoulos, K.N.; Philp, A.; Hengist, A., et al. Lipid metabolism links nutrient-exercise timing to insulin sensitivity in men classified as overweight or obese. *J. Clin. Endocrinol Metab* **2019**, 10.1210/clinem/dgz104, doi:10.1210/clinem/dgz104.
103. Currell, K.; Jentjens, R.L.; Jeukendrup, A.E. Reliability of a cycling time trial in a glycogen-depleted state. *Eur J. Appl Physiol* **2006**, *98*, 583–589, doi:10.1007/s00421-006-0305-7.
104. Podlogar, T.; Free, B.; Wallis, G.A. High rates of fat oxidation are maintained after the sleep low approach despite delayed carbohydrate feeding during exercise. *Eur J. Sport Sci* **2020**, 10.1080/17461391.2020.1730447, 1-11, doi:10.1080/17461391.2020.1730447.
105. Paris, H.L.; Fulton, T.J.; Wilhite, D.P.; Baranauskas, M.N.; Chapman, R.F.; Mickleborough, T.D. "Train-High Sleep-Low" Dietary Periodization Does Not Alter Ventilatory Strategies During Cycling Exercise. *J. Am. Coll Nutr* **2020**, *39*, 325–332.
106. Wee, S.-L.; Williams, C.; Tsintzas, K.; Boobis, L. Ingestion of a high-glycemic index meal increases muscle glycogen storage at rest but augments its utilization during subsequent exercise. *J. Appl Physiol* **2005**, *99*, 707–714.
107. Wong, S.H.; Siu, P.M.; Lok, A.; Chen, Y.; Morris, J.; Lam, C. Effect of the glycaemic index of pre-exercise carbohydrate meals on running performance. *Eur J. Sport Sci* **2008**, *8*, 23–33.

108. Wu, C.-L.; Williams, C. A low glycemic index meal before exercise improves endurance running capacity in men. *International journal of sport nutrition and exercise metabolism* **2006**, *16*, 510–527.
109. Bartlett, J.D.; Louhelainen, J.; Iqbal, Z.; Cochran, A.J.; Gibala, M.J.; Gregson, W.; Close, G.L.; Drust, B.; Morton, J.P. Reduced carbohydrate availability enhances exercise-induced p53 signaling in human skeletal muscle: implications for mitochondrial biogenesis. *Am. J. Physiol Regul Integr Comp. Physiol* **2013**, *304*, R450–R458, doi:10.1152/ajpregu.00498.2012.
110. Akerstrom, T.C.; Birk, J.B.; Klein, D.K.; Erikstrup, C.; Plomgaard, P.; Pedersen, B.K.; Wojtaszewski, J. Oral glucose ingestion attenuates exercise-induced activation of 5'-AMP-activated protein kinase in human skeletal muscle. *Biochem Biophys Res. Commun* **2006**, *342*, 949–955, doi:10.1016/j.bbrc.2006.02.057.
111. Birk, J.B.; Wojtaszewski, J.F. Predominant alpha2/beta2/gamma3 AMPK activation during exercise in human skeletal muscle. *J. Physiol* **2006**, *577*, 1021–1032, doi:10.1113/jphysiol.2006.120972.
112. Clark, S.A.; Chen, Z.-P.; Murphy, K.T.; Aughey, R.; McKenna, M.; Kemp, B.E.; Hawley, J.A. Intensified exercise training does not alter AMPK signaling in human skeletal muscle. *Am. J. Physiol Endocrinol Metab* **2004**, *286*, E737–E743.
113. Edinburgh, R.M.; Hengist, A.; Smith, H.A.; Travers, R.L.; Koumanov, F.; Betts, J.A.; Thompson, D.; Walhin, J.P.; Wallis, G.A.; Hamilton, D.L., et al. Preexercise breakfast ingestion versus extended overnight fasting increases postprandial glucose flux after exercise in healthy men. *Am. J. Physiol Endocrinol Metab* **2018**, *315*, E1062–E1074, doi:10.1152/ajpendo.00163.2018.
114. Lee-Young, R.S.; Palmer, M.J.; Linden, K.C.; LePlastrier, K.; Canny, B.J.; Hargreaves, M.; Wadley, G.D.; Kemp, B.E.; McConell, G.K. Carbohydrate ingestion does not alter skeletal muscle AMPK signaling during exercise in humans. *Am. J. Physiol Endocrinol Metab* **2006**, *291*, E566–E573.
115. Wadley, G.D.; Lee-Young, R.S.; Canny, B.J.; Wasuntarawat, C.; Chen, Z.P.; Hargreaves, M.; Kemp, B.E.; McConell, G.K. Effect of exercise intensity and hypoxia on skeletal muscle AMPK signaling and substrate metabolism in humans. *Am. J. Physiol Endocrinol Metab* **2006**, *290*, E694–E702, doi:10.1152/ajpendo.00464.2005.
116. McConell, G.K.; Lee-Young, R.S.; Chen, Z.P.; Stepto, N.K.; Huynh, N.N.; Stephens, T.J.; Canny, B.J.; Kemp, B.E. Short-term exercise training in humans reduces AMPK signalling during prolonged exercise independent of muscle glycogen. *J. Physiol* **2005**, *568*, 665–676, doi:10.1113/jphysiol.2005.089839.
117. Mortensen, B.; Hingst, J.R.; Frederiksen, N.; Hansen, R.W.; Christiansen, C.S.; Iversen, N.; Friedrichsen, M.; Birk, J.B.; Pilegaard, H.; Hellsten, Y., et al. Effect of birth weight and 12 weeks of exercise training on exercise-induced AMPK signaling in human skeletal muscle. *Am. J. Physiol Endocrinol Metab* **2013**, *304*, E1379–E1390, doi:10.1152/ajpendo.00295.2012.
118. Nielsen, J.N.; Mustard, K.J.; Graham, D.A.; Yu, H.; MacDonald, C.S.; Pilegaard, H.; Goodyear, L.J.; Hardie, D.G.; Richter, E.A.; Wojtaszewski, J.F. 5'-AMP-activated protein kinase activity and subunit expression in exercise-trained human skeletal muscle. *J. Appl Physiol (1985)* **2003**, *94*, 631–641, doi:10.1152/japplphysiol.00642.2002.
119. Roepstorff, C.; Thiele, M.; Hillig, T.; Pilegaard, H.; Richter, E.A.; Wojtaszewski, J.F.; Kiens, B. Higher skeletal muscle α2AMPK activation and lower energy charge and fat oxidation in men than in women during submaximal exercise. *The Journal of physiology* **2006**, *574*, 125–138.
120. Wojtaszewski, J.F.; Nielsen, P.; Hansen, B.F.; Richter, E.A.; Kiens, B. Isoform-specific and exercise intensity-dependent activation of 5'-AMP-activated protein kinase in human skeletal muscle. *J. Physiol* **2000**, *528 Pt. 1*, 221-226, doi:10.1111/j.1469-7793.2000.t01-1-00221.x.
121. Yu, M.; Stepto, N.K.; Chibalin, A.V.; Fryer, L.G.; Carling, D.; Krook, A.; Hawley, J.A.; Zierath, J.R. Metabolic and mitogenic signal transduction in human skeletal muscle after intense cycling exercise. *J. Physiol* **2003**, *546*, 327–335, doi:10.1113/jphysiol.2002.034223.
122. Civitarese, A.E.; Hesselink, M.K.; Russell, A.P.; Ravussin, E.; Schrauwen, P. Glucose ingestion during exercise blunts exercise-induced gene expression of skeletal muscle fat oxidative genes. *Am. J. Physiol Endocrinol Metab* **2005**, *289*, E1023–E1029, doi:10.1152/ajpendo.00193.2005.
123. Fujii, N.; Hayashi, T.; Hirshman, M.F.; Smith, J.T.; Habinowski, S.A.; Kaijser, L.; Mu, J.; Ljungqvist, O.; Birnbaum, M.J.; Witters, L.A., et al. Exercise induces isoform-specific increase in 5'AMP-activated protein kinase activity in human skeletal muscle. *Biochem Biophys Res. Commun* **2000**, *273*, 1150–1155, doi:10.1006/bbrc.2000.3073.

124. Stephens, T.J.; Chen, Z.P.; Canny, B.J.; Michell, B.J.; Kemp, B.E.; McConell, G.K. Progressive increase in human skeletal muscle AMPKalpha2 activity and ACC phosphorylation during exercise. *Am. J. Physiol Endocrinol Metab* **2002**, *282*, E688–E694, doi:10.1152/ajpendo.00101.2001.
125. Treebak, J.T.; Pehmoller, C.; Kristensen, J.M.; Kjobsted, R.; Birk, J.B.; Schjerling, P.; Richter, E.A.; Goodyear, L.J.; Wojtaszewski, J.F. Acute exercise and physiological insulin induce distinct phosphorylation signatures on TBC1D1 and TBC1D4 proteins in human skeletal muscle. *J. Physiol* **2014**, *592*, 351–375, doi:10.1113/jphysiol.2013.266338.
